# Magnetic Circuit Design and Optimization of Tension–Compression Giant Magnetostrictive Force Sensor

**DOI:** 10.3390/s26010295

**Published:** 2026-01-02

**Authors:** Long Li, Hailong Sun, Yingling Wei, Boda Li, Hongwei Cui, Ruifeng Liu

**Affiliations:** 1 College of Aeronautics and Astronautics, Taiyuan University of Technology, Taiyuan 030024, China; lilong01@tyut.edu.cn (L.L.);; 2 College of Mechanical Engineering, Taiyuan University of Technology, Taiyuan 030024, China; 3 College of Chemistry and Chemical Engineering, Taiyuan University of Technology, Taiyuan 030024, China; 4 College of Civil Engineering, Taiyuan University of Technology, Taiyuan 030024, China

**Keywords:** giant magnetostrictive force sensor, magnetic conductive materials, magnetic circuit, uniformity, COMSOL finite element simulation

## Abstract

The variable-pitch connecting rod of a helicopter bears axial tensile and compressive loads during operation. The traditional load monitoring method using strain gauge is easily affected by external conditions. Therefore, a giant magnetostrictive (GM) tension and compression force sensor with permanent magnet bias is proposed and optimized. Because the bias magnetic field plays a decisive role in the performance of the sensor, this paper has carried out in-depth research on this. Firstly, the mathematical model of the magnetic circuit is established, and the various magnetic circuits of the sensor are simulated and analyzed. Secondly, the magnetic flux uniformity of the GMM rod is used as the evaluation index, and the relative permeability of the magnetic material and the structure are systematically studied. The influence of parameters on the magnetic flux of the magnetic circuit, and finally the optimal parameter combination of the magnetic circuit is determined by orthogonal test. The results show that when the magnetic circuit without the magnetic side wall is used, the magnetic material can better guide the magnetic flux through the GMM rod; the magnetic flux uniformity of the optimized GMM force sensor is increased by 7.44%, the magnetic flux density is increased by 13.9 mT and the Hall output voltage increases linearly by 1.125% in the same proportion. This provides an important reference for improving the utilization rate of GMM rods and also improves the safety of flight operation and reduces maintenance costs.

## 1. Introduction

Helicopter rotor load monitoring is an important way to improve the safety and reliability of aircraft operation. The traditional load monitoring method uses strain gauges. The rotor surface needs to paste a large number of strain gauges. A large number of strain gauges will change the aerodynamic shape of the rotor blades and affect the aerodynamic efficiency and aeroelastic response characteristics of the rotor [1]. With the continuous progress and innovation of technology, it is necessary to promote new load monitoring methods in a timely manner.

Giant magnetostrictive material is a new type of intelligent material with high Curie temperature, high magneto mechanical coupling coefficient, good frequency response characteristics and large magnetostrictive strain, which makes it widely studied in the fields of energy conversion, micro drive, fluid machinery, precision machining, linear motor, vibration control, ultrasonic and so on [2,3,4,5]. Yan and Yang gave the design principle of the giant magnetostrictive force sensor based on the analysis of the magnetostrictive inverse effect and designed the structure of the giant magnetostrictive force sensor [6]. Liu developed a giant magnetostrictive force sensing actuator with a powerful sensing and controllable output force [7]. In addition to the basic executive functions of output displacement and force, it also has the functions of force measurement, output force sensing and controllable output force. Soheil Talebian [8] mentioned that the geometric shape, magnetic circuit design and working conditions of magnetostrictive materials will have a certain impact on the sensor output. Li et al. designed a differential resonant magnetic field sensor, which includes a compact magnetic circuit with a pair of permanent magnets and double FeGa plates to ensure differential drive, which not only reduces the leakage flux, but also improves the field sensitivity and resolution [9]. Wang et al. systematically studied the influence of magnetizer parameters on magnetic flux density and eddy current [10]. The average magnetic flux density after optimization in the static magnetic field increased by 0.0161 T, and the uniformity increased by 4.5%. Liu et al. optimized the magnetic induction intensity on the center line of the GMM rod by 0.18 T, and the uniformity increased to 92% [11]. However, they only analyzed the upper magnetic conductive material and did not analyze the overall magnetic circuit. Tu [12] proposed the principle of uniformity and optimized the magnetic circuit of the giant magnetostrictive brake. The magnetic induction intensity through the magnetic circuit increased by 0.1 T, and the uniformity increased by 10.27%. For these studies, there is no systematic study of the influence of structural parameters on the flux of GMM rods.

There are two ways to provide bias magnetic field: DC bias and permanent magnet bias. DC bias requires additional DC power supply. In contrast, permanent magnet bias does not require additional power supply and can provide a more stable bias magnetic field [13,14]. In addition, there are two main ways to bias permanent magnets: external tubular permanent magnets and stacked permanent magnets [15,16]. Because the force sensor needs to be subjected to a large force, the permanent magnet has a weak ability to bear the load, so the external tubular permanent magnet is more suitable for the large load GM force sensor to achieve.

The GMM rod is the core of the whole sensor, and the main indicators to measure its performance include the magnetic field uniformity and magnetic flux intensity through the GMM rod. In this paper, based on the principle of improving the magnetic flux density uniformity and magnetic induction intensity of GMM rod, the influence of the arrangement of magnetic materials on the magnetic flux uniformity and magnetic flux density of magnetic circuit is analyzed under the condition of using permanent magnet to provide bias magnetic field. The influence of the internal parameters of magnetic materials and the structure size of magnetic materials on the magnetic flux uniformity and magnetic flux density of GMM rod is studied.

## 2. Structure Design of Giant Magnetostrictive Force Sensor

**Figure 1:**
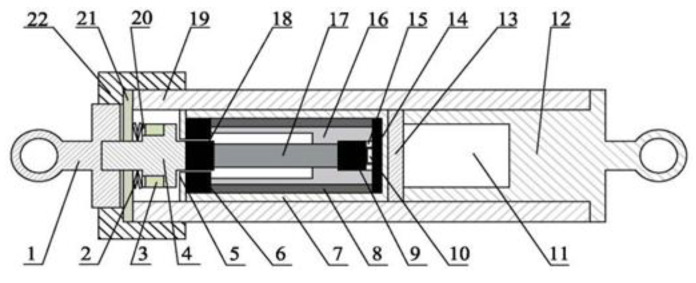
Rotor load monitoring sensor structure diagram. 1—upper bearing (input shaft); 2—disk spring; 3—piezoelectric ceramics; 4—force transfer shaft; 5—magnetic isolation end cover; 6—upper conducting magnetic sheet; 7—magnetic isolation sleeve; 8—permanent magnet; 9—lower conducting magnetic block; 10—Hall effect sensor; 11—wireless acquisition and radio frequency emission module and energy harvesting module integrated circuit board; 12—lower end bearing; 13—magnetostrictive force sensor baffle; 14—lower conductive magnetic sheet; 15—stainless steel sleeve; 16—fixed frame; 17—GMM rod; 18—upper conducting magnetic block; 19-variable-pitch connecting rod barrel wall; 20—piezoelectric ceramic lead sleeve; 21—piezoelectric ceramic cover plate; 22—barrel wall cover.

First, we will introduce the overall working principle of the sensor. The load monitoring sensor is embedded and installed inside the variable-pitch connecting rod. It consists of three parts: energy harvester (component 3), magnetostrictive load sensor (components 6–10 and 14–18), data acquisition and wireless RF transmission (component 11). The energy harvester collects the energy generated by the piezoelectricity to supply power for the magnetostrictive load sensor and the data acquisition and wireless RF transmission. The data acquisition and wireless RF transmission module collects the voltage signal of the magnetostrictive load sensor. The specific structure diagram is shown in Figure 1.

The upper bearing and the force transfer shaft are connected by thread, which is convenient for the piezoelectric ceramic, the disk spring, the piezoelectric ceramic lead sleeve and the piezoelectric ceramic cover plate to penetrate into it. Components 1, 2 and 4 form the input part of the sensor load, which transmits the load along the axial direction of the GMM rod to the GMM rod. The load input part is subjected to the interaction between the dynamic load and the disk spring, which can realize the continuous transmission of the axial load. It can apply dynamic load to the piezoelectric ceramic and the GMM rod, which not only ensures that the energy collection module can always generate voltage during the working process, but also enables the force sensor module to monitor the change in load. In addition, the variable-pitch connecting rod barrel wall and the barrel wall cover plate are connected by thread. By adjusting the matching distance between the two, the compression amount of the disk spring can be indirectly changed, and then the preload of the GMM rod and its deformation can be adjusted. The lower end bearing and the variable-pitch connecting rod barrel wall are connected by thread, which leaves enough space for the upper force sensor module and the piezoelectric module, and ensures that the circuit module can work stably and reliably.

The giant magnetostrictive force sensor is the core component of the entire sensor, and the article also focuses on this part for research. This part of the component can realize the dynamic load measurement along the axial direction of GMM rod. The structure diagram of the giant magnetostrictive force sensor is shown in Figure 1, which is mainly composed of the GMM rod, permanent magnet, fixed frame, upper and lower conducting magnetic blocks, upper and lower conducting magnetic sheets, stainless steel sleeve, Hall effect sensor, disk spring and magnetostrictive force sensor baffle. The compressive strength of the GMM is higher, while the tensile strength is lower. The disk spring can provide a certain pressure for the GMM rod. By monitoring the force of the GMM rod, the tension and pressure of the variable-pitch connecting rod are indirectly monitored. The magnetic circuit is composed of the upper and lower magnetic sheets, the upper and lower conducting magnetic blocks, the GMM rod and the permanent magnet, so that the magnetic flux can pass through the GMM rod better. The Hall element monitors the variation in the magnetic field and indirectly reflects the magnitude of the external force on the GMM rod through the output voltage. Because the force of the Hall element will affect its performance and even lead to the damage of the Hall element, the Hall element can be placed inside a stainless steel sleeve to protect the Hall element. In this design, the way of using the coil to provide the bias magnetic field is changed, but the permanent magnet is used to provide the bias magnetic field.

Neodymium-iron-boron (NdFeB) currently boasts the highest magnetic energy product among commercialized magnets. It can operate within a temperature range from −40 °C to 80 °C. The temperature tolerance of the external fuselage of the aircraft is generally in the range from −40 °C to +50 °C. Additionally, due to its high magnetic energy product, the volume of permanent magnets can be reduced in design, thereby minimizing the structure of sensors and enabling their application in numerous fields involving micro-precision force monitoring.

## 3. Theoretical Model

The permanent magnet is used as the bias magnetic field to provide the excitation source for the force sensor. Based on the theory of piezomagnetic effect and through a series of derivations, the relationship between the magnetic field and the geometric structure of the sensor is obtained. The piezomagnetic equations shown in Equations (1) and (2) can be obtained, which represent the relationship between magnetostrictive phenomenon and mechanical stress and magnetic field [17]:

(1) $$\varepsilon =S^{H}\sigma +\lambda H$$

(2) $$B_{G}=g\sigma +\mu^{\sigma }H$$

where *λ* is magnetostriction coefficient, *σ* is the stress of GMM rod, *ε* is the strain of GMM rod under certain stress, *H* is the average magnetic field intensity through GMM rod, *B_G_* is the average magnetic flux density through GMM rod, *S^H^* is the elastic coefficient of GMM under constant temperature and constant magnetic field, g is the piezomagnetic coefficient and *μ^σ^* is the permeability of GMM rod under certain stress.

The bias magnetic field provided by the permanent magnet is stable. There is a proportional coefficient between the magnetic flux density of the Hall element and the GMM rod, and the coefficient is almost not affected by the external load [18]. Assuming that the proportional coefficient is C, Equation (3) can be obtained:

(3) $$\frac{B_{1}}{B_{G}}=C$$

where *B*_1_ is the average magnetic field density through the Hall element.

The relationship between the output voltage of the Hall element and the magnetic flux density passing through it is shown in Equation (4).

(4) $$E=q\frac{IB_{1}}{d}+U_{0}$$

where *E* is the output voltage of the Hall element, q is the sensitivity coefficient, *I* is the current intensity, *d* is the thickness of the Hall element and U_0_ is the base voltage.

The relationship between magnetic flux and stress–strain:

(5) $$B_{G}\approx \sigma \left(g-\frac{s^{H}\mu^{\sigma }}{\lambda }\right)+\frac{\mu^{\sigma }B_{0}^{2}}{\lambda E\mu_{0}}$$

Here, *B*_0_ refers to the average magnetic flux density (T) through GMM in the unstressed state. A control model based on magnetic induction intensity:

(6) $$\lambda \approx \frac{\Delta L}{L}\approx \frac{{B_{0}}^{2}}{E\mu_{0}}$$

It can be seen from Equation (5) that when the stress of the GMM rod is constant, the magnetic flux density through the GMM rod is linearly related to the initial magnetic flux density through the GMM rod.

**Figure 2:**
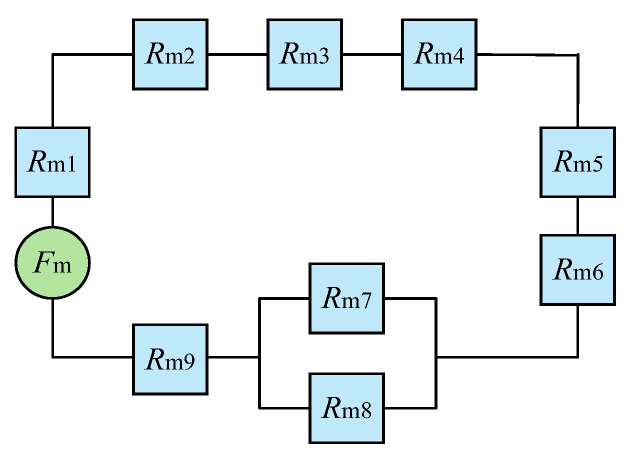
Summary of the model of the giant magnetostrictive force sensor, where *F*_m_ is the total magnetomotive force generated by the permanent magnet, *R*_m1_ to *R*_m9_ represent the equivalent magnetoresistances of permanent magnet, upper conducting magnetic sheet, upper air gap, upper conducting magnetic block, GMM rod, lower conducting magnetic block, stainless steel sleeve, air gap and lower conducting magnetic sheet, respectively. Similarly, in subsequent models, *μ*_m1_ to *μ*_m9_, *S*_m1_ to *S*_m9_ and *L*_m1_ to *L*_m9_ represent relative permeability, cross-sectional area and height, respectively.

Figure 2 shows the model simplification diagram for the force sensor. In view of the magnetic circuit formed by the surrounding high permeability materials, the magnetic field passes through each component in turn to form a series relationship. At the same time, at the interface between the lower magnetic block and the stainless steel layer, the magnetic field will pass through the stainless steel and its internal air gap in parallel, and it is determined that *R*_m7_ and *R*_m8_ are in parallel.

The total magnetoresistance of the magnetic circuit is:

(7) $$R_{m}=R_{m1}+R_{m2}+R_{m3}+R_{m4}+R_{m5}+R_{m6}+R_{m9}+\frac{R_{m7}R_{m8}}{R_{m7}+R_{m8}}$$

Because the permeability of the GMM rod and permanent magnet is low, the magnetic flux leakage should be considered in the actual magnetic circuit. When the magnetic flux leakage is not considered, the magnetomotive force generated by the permanent magnet is assumed to be *F*_mn_, and the magnetic flux leakage coefficient of the permanent magnet is defined by *K*_t_, which can be taken as 1.1–1.3 [19]. The magnetomotive force of the permanent magnet can be calculated by the following equation according to the magnetic circuit Ohm’s law [20]:

(8) $$F_{mn}=\frac{B_{r}L_{m1}}{\mu_{m1}\mu_{0}}$$

(9) $$F_{m}=\frac{B_{r}L_{m1}}{\mu_{m1}\mu_{0}K_{t}}$$

where *F*_m_ represents the magnetomotive force of the permanent magnet in the case of magnetic flux leakage; *B*_r_ is the residual magnetic induction intensity of the permanent magnet.

According to the magnetic circuit Ohm’s law, the initial magnetic flux through the GMM rod can be expressed in Equation (10):

(10) $$B_{0}=\frac{\phi }{S_{m5}}=\frac{F_{m}}{R_{m}S_{m5}}$$

Equation (11) can be obtained by combining Equations (5) and (7):

(11) $$B_{G}\approx \sigma \left(g-\frac{s^{H}\mu^{\sigma }}{\lambda }\right)+\frac{\mu^{\sigma }}{\lambda E\mu_{0}}\times \left(\frac{F_{m}}{R_{m}S_{m5}}\right)$$

From Equations (7) and (9), we can obtain Equation (12):

(12) $$B_{G}\approx \sigma \left(g-\frac{s^{H}\mu^{\sigma }}{\lambda }\right)+\frac{\mu^{\sigma }}{\lambda E\mu_{0}}\times \left(\frac{F_{m}}{\left(R_{m1}+R_{m2}+R_{m3}+R_{m4}+R_{m5}+R_{m6}+R_{m9}+\frac{R_{m7}R_{m8}}{R_{m7}+R_{m8}}\right)S_{m5}}\right)$$

Through Equation (12), it can be seen that the magnetic flux density of the GMM rod is related to the thickness and radius of the magnetic material and the size of the air gap. Therefore, it is necessary to optimize the sensor model by increasing the magnetic flux density through the GMM rod.

## 4. Based on COMSOL Multiphysics Finite Element Simulation Analysis

### 4.1. Finite Element Model of Giant Magnetostrictive Force Sensor

**Figure 3:**
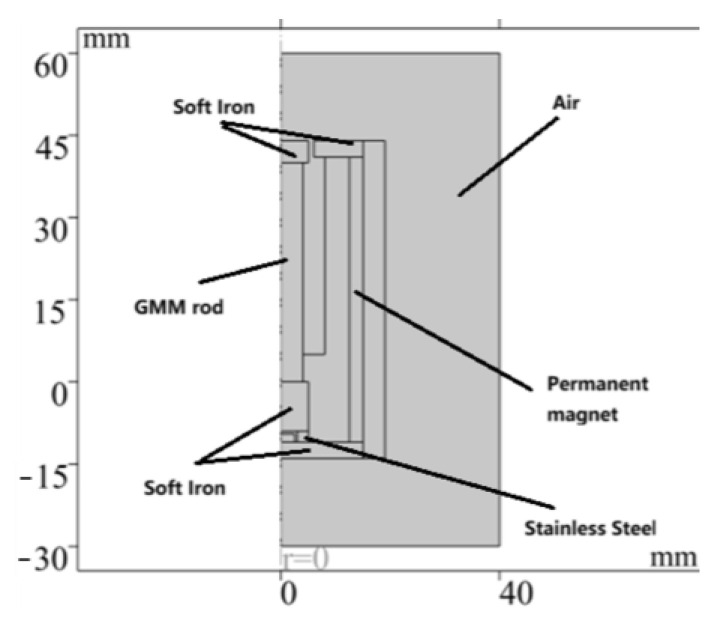
Finite element model.

**Figure 4:**
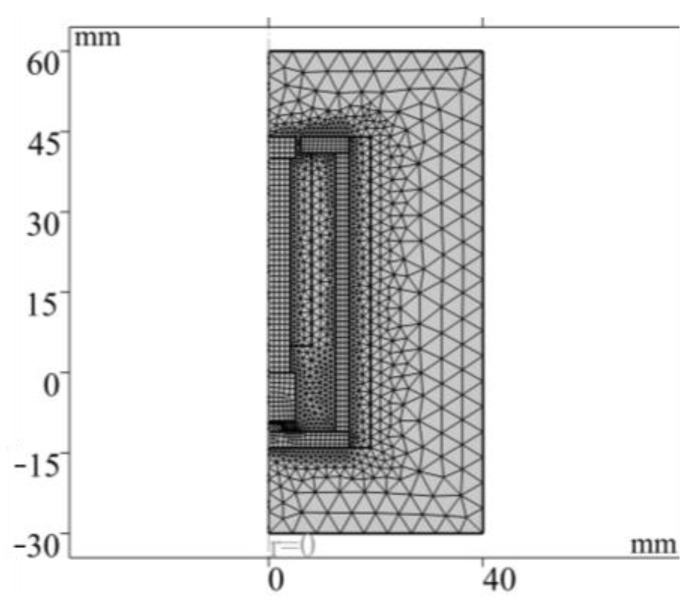
Grid division.

Since the GM force sensor is an axisymmetric geometric structure, it can be simplified to a 2D plane model for finite element analysis and calculation. The magnetic isolation end cover, magnetic isolation sleeve, fixture and preload spring have little influence on the magnetic flux of the whole magnetic circuit, so they are ignored in the simulation analysis. The finite element simulation analysis process using COMSOL Multiphysics 6.1 is as follows:

(1) Establish a two-dimensional axisymmetric model in geometry, as shown in Figure 3.

(2) Define the material properties of the relevant material, as shown in Table 1. Select the domain corresponding to the material.

(3) The boundary conditions, magnetic circuit and air domain are set and meshed. The results are shown in Figure 4.

(4) The simulation process uses the orthogonal method to analyze the magnetic circuit and output the simulation results through the drawing group.

The established finite element model (Figure 3) corresponds to the sensor design in Figure 1. The material parameters of each component are listed in Table 1. In order to ensure calculation accuracy, the local mesh refinement of the air gap region is carried out, and the final generated model contains a total of 1619 elements and 1052 nodes (Figure 4). Finally, the numerical simulation of the model is completed by using the steady-state MUMPS solver.

### 4.2. Effect of Magnetic Circuit Mode on GMM Flux

The GMM rod is the core of the force sensor. The different arrangements of the magnetic material will have different effects on the magnetic flux of the magnetic circuit. Therefore, it is necessary to analyze the magnetic material of the magnetic circuit in various situations. The following eight different magnetic circuit structures are designed (a. has upper and lower magnetic materials without magnetic side walls; b. no upper and lower magnetic materials and no magnetic side wall; c. has a lower magnetic material without a magnetic side wall; d. has upper magnetic material without magnetic side wall; e. has a lower magnetic material with a magnetic side wall; f. has upper magnetic material with magnetic side wall; g. has upper and lower magnetic conductive materials with magnetic conductive side walls; h. there is no upper and lower magnetic material with magnetic side wall). The finite element simulation analysis of these 8 kinds of magnetic circuit structures is carried out, and the comparison diagram of magnetic flux density at the center line of the GMM rod is obtained.

Taking the uniformity of magnetic field distribution as the evaluation standard of magnetic circuit [21], the expression is

(13) $$\eta =\left(1-\frac{B_{\max}-B_{\min}}{B_{\max}}\right)\times 100\%$$

where *B*_max_ refers to the maximum magnetic flux density through the central axis of the giant magnetostrictive material (GMM) rod, and *B*_min_ denotes the minimum magnetic flux density through the central axis of the GMM rod.

Note: In order to facilitate the simulation and avoid the repeated setting of the magnetic circuit materials, in the above analysis of a variety of magnetic circuits, the upper and lower magnetic materials and the magnetic side walls are not set according to the relevant parameters of the air. The centerline magnetic flux density of the GMM rod is represented by B_2_. The surface average magnetic flux density of the GMM rod is represented by B_3_.

**Figure 5:**
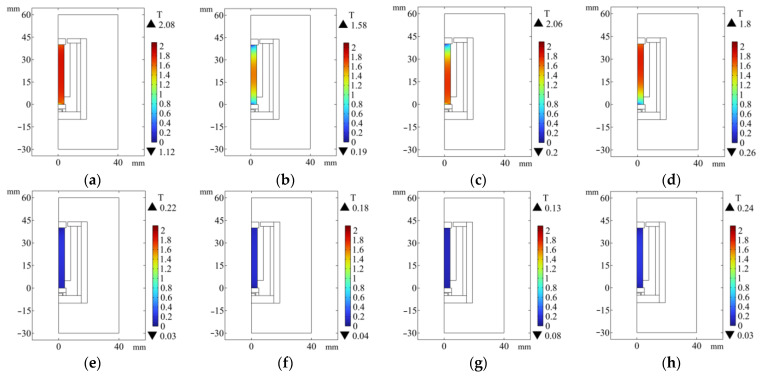
Magnetic flux density diagram with or without magnetic material and magnetic sidewall surface. (**a**) With upper and lower magnetic conductive materials and no magnetic conductive side walls; (**b**) Without upper and lower magnetic conductive materials and without magnetic conductive side walls; (**c**) With lower magnetic conductive material and no magnetic conductive side walls; (**d**) With upper magnetic conductive material and no magnetic conductive side walls; (**e**) With lower magnetic conductive material and magnetic conductive side walls; (**f**) With upper magnetic conductive material and magnetic conductive side walls; (**g**) With upper and lower magnetic conductive materials and magnetic conductive side walls; (**h**) Without upper and lower magnetic conductive materials and magnetic conductive side walls.

**Figure 6:**
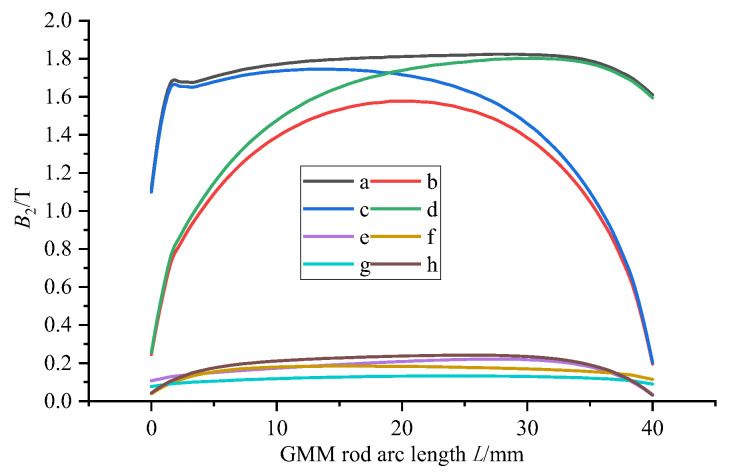
Comparison of GMM magnetic flux.

It can be seen from Figure 5 that the color of the magnetic flux density cloud of several GMM rods without the magnetic side wall is deeper than that with the magnetic side wall. It can also be seen from Figure 6 that the magnetic flux through the GMM rod can be improved by using the non-magnetic side wall compared with the magnetic side wall, and the magnetic flux of the GMM rod is more uniform. The analysis of several cases without magnetic side walls shows that the upper magnetic material has a great influence on the magnetic flux at the upper end of the GMM rod, while the lower magnetic material has a great influence on the magnetic flux at the lower end of the GMM rod. When the upper and lower magnetic materials have no magnetic side wall, the maximum and minimum values of the magnetic flux density surface are larger than other methods and the magnetic flux is more uniform. Therefore, the magnetic circuit structure of the upper and lower magnetic materials without the magnetic side wall can improve the magnetic flux through the GMM rod to be larger and more uniform.

### 4.3. Effect of Relative Permeability of Magnetic Material on Magnetic Flux of Magnetic Circuit

**Figure 7:**
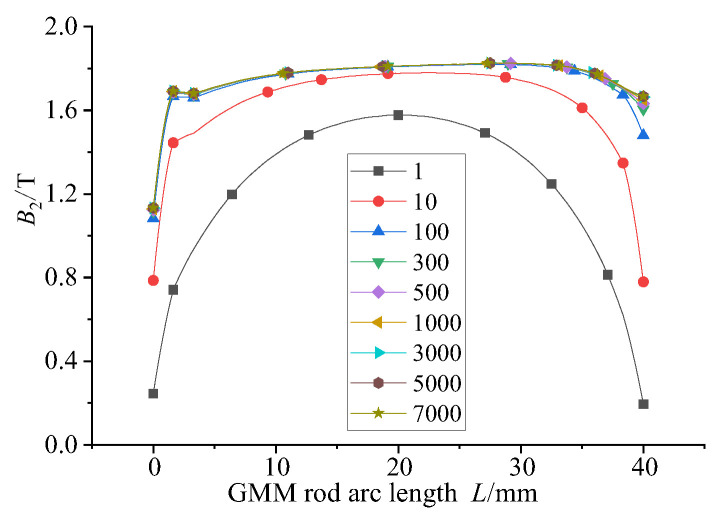
GMM centerline magnetic flux density distribution.

**Figure 8:**
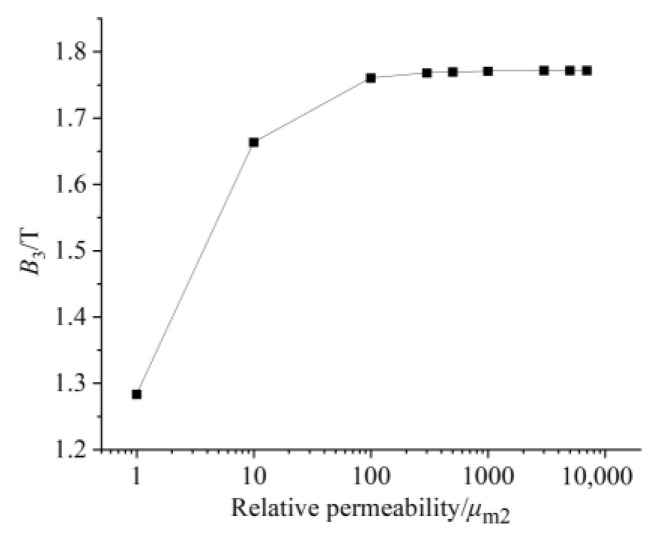
Relationship between GMM magnetic flux and relative permeability.

**Figure 9:**
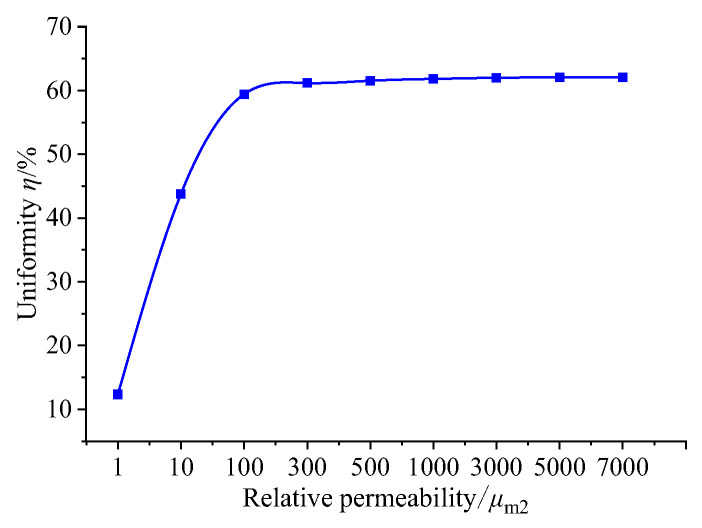
The relationship between uniformity and relative permeability.

**Figure 10:**
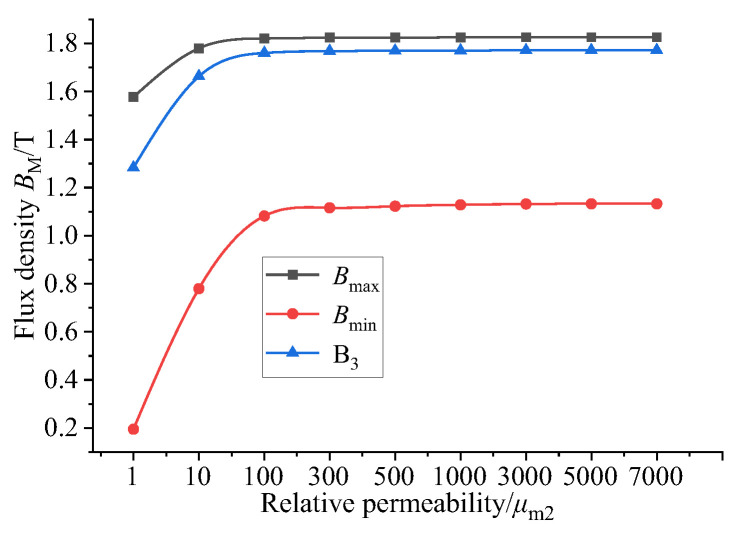
The relationship between the maximum and minimum magnetic flux density and relative permeability.

In the magnetic circuit, the relative permeability of the GMM rod is low. The relative permeability of the magnetic material that completes the magnetic circuit coupling determines the ability of the material to transfer the magnetic flux, thus affecting the magnetic flux distribution, magnetic flux density uniformity and magnetic flux density of the magnetic circuit. The relative permeability of the upper and lower magnetizers and the magnetic sheet is set to 1, 10, 100, 300, 500, 1000, 3000, 5000 and 7000. The magnetic flux density at the center line of GMM is selected as the research object. The results are as follows:

By observing Figure 7 and Figure 8, it can be seen that when the relative permeability is 1, the magnetic induction intensity of the center line of the GMM rod is small as a whole. At this time, the magnetic flux leakage phenomenon is more serious. The magnetic induction intensity of the center line of the GMM rod increases with the increase in relative permeability. When the relative permeability is 3000, 5000 and 7000, the central magnetic induction intensity curve of GMM is coincident. It can be seen from Figure 9 that when the relative permeability is 1, the magnetic induction intensity of the GMM center line is 12.34%. With the increase in the relative permeability, uniformity also increases gradually. When the relative permeability reaches 3000, the uniformity is 62.01%, and at permeabilities 5000 and 7000, it tends to remain unchanged compared with the relative permeability of 3000. It can also be seen from Figure 10 that with the increase in the relative permeability of the magnetic material, the maximum and minimum values of the magnetic flux density are consistent with the uniformity of the surface average magnetic flux density, which increases first and then tends to remain unchanged.

In summary, when designing the magnetic material, the magnetic material with larger permeability should be selected, which can improve the magnetic flux uniformity and magnetic flux density of the GMM rod.

### 4.4. The Influence of Structural Parameters of Magnetic Materials on the Magnetic Flux of Magnetic Circuit

Magnetic material with a relative permeability of 4000 is used for the following research. The GMM magnetic flux conduction mainly depends on the upper and lower magnetic materials supporting the GMM rod. It can be seen from the model diagram that the lower conducting magnetic sheet is not in contact with the GMM rod, and the remaining magnetic material is in contact with the GMM rod. The thickness of the magnetic material can affect the magnetic flux density and uniformity of the GMM rod, so it is necessary to select the appropriate thickness of the magnetic material. Firstly, only the thickness of the lower conducting magnetic sheet is changed under the condition that the size of other magnetic materials is unchanged. The thickness of the lower conducting magnetic sheet is set to 1~8 mm, and the step size is 1 mm. The following figure can be obtained by simulation in COMSOL.

**Figure 11:**
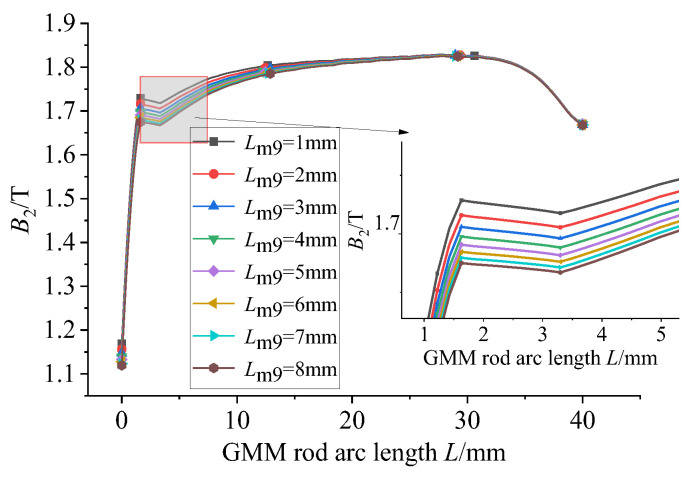
*B*_3_ under the influence of the thickness of the lower magnetic sheet.

**Figure 12:**
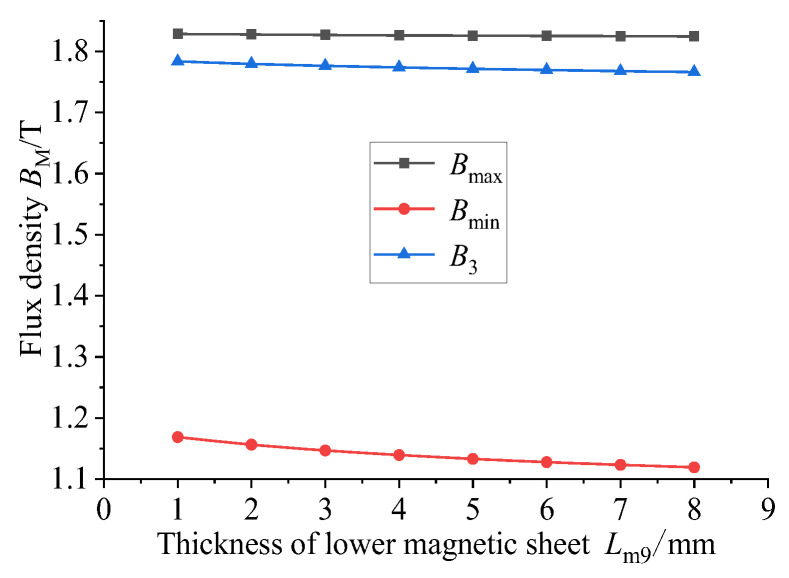
The magnetic flux density under different thicknesses of the magnetic sheet.

**Figure 13:**
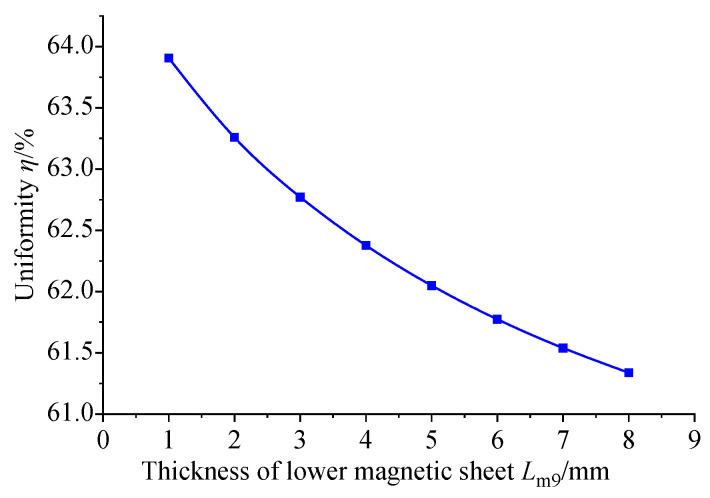
The magnetic flux uniformity of the GMM rod center line under the influence of the thickness of the lower magnetizer.

Figure 11 shows the comparison of the magnetic flux density of the GMM center line under the condition that the thickness of the upper conducting magnetic sheet is 4 mm, the thickness of the lower conducting magnetic sheet is 3 mm and the thickness of the lower conducting magnetic sheet is changed. It can be seen that as the thickness of the lower conducting magnetic sheet increases, the magnetic flux density of the GMM center line gradually decreases, and the trend gradually slows down. The magnetic flux density diagram shown in Figure 12 is consistent with the trend shown in Figure 11. The uniformity diagram shown in Figure 13 also decreases with the increase in the thickness of the lower magnetic sheet, and the deceleration gradually slows down.

**Figure 14:**
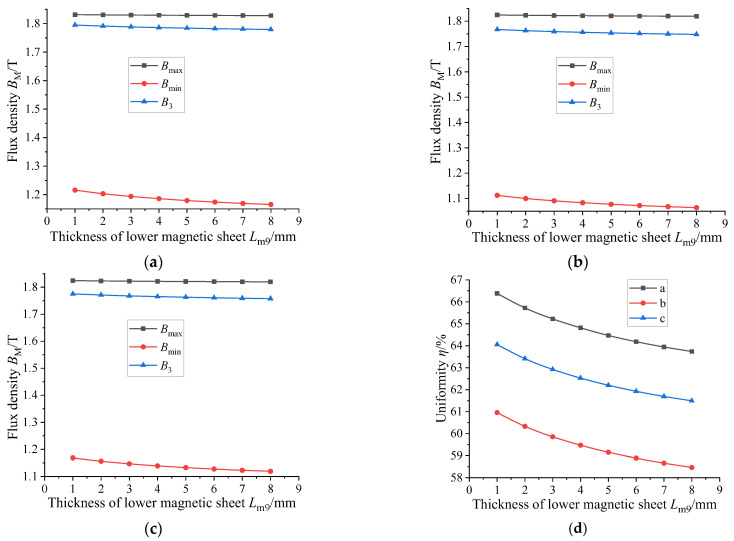
The influence of the thickness of the magnetic sheet under different conditions.

In order to verify the universality of the law in the above cases and to examine the effect of increasing or decreasing the thickness of the upper conducting magnetic block and the lower conducting magnetic block, three configurations were adopted: (a) upper conducting magnetic block 5 mm, lower conducting magnetic block 4 mm; (b) 3 mm upper conducting magnetic block, 2 mm lower conducting magnetic block; (c) the upper conducting magnetic block is 3 mm, and the lower conducting magnetic block is 3 mm; (d) thickness uniformity of different upper magnetic blocks. The range of the lower conducting magnetic sheet is 1~8 mm, and the step size is 1 mm. By comparing the above three cases, Figure 14 can be obtained by calculating the uniformity:

From the three figures of (a), (b) and (c) in Figure 14, it can be seen that the maximum, minimum and surface average values of magnetic flux density in the three cases are the same as those in Figure 11. In addition, the trend of magnetic flux uniformity in Figure 14d is the same as that in Figure 13.

Considering the actual situation, the thickness of the conducting magnetic sheet is too small to be processed, the processing cost will increase and the thickness is too large to increase the structure. At the same time, it will also reduce the uniformity and magnetic flux density of the GMM rod, resulting in the weakening of the performance of the sensor. Therefore, it is more appropriate to set the thickness of the lower conducting magnetic sheet to about 3 mm. Based on the thickness of the lower conducting magnetic sheet of 3 mm, the influence of the thickness of the upper conducting magnetic sheet, the upper conducting magnetic sheet and the lower conducting magnetic sheet on the uniformity of the magnetic flux density of the center line of the GMM rod and the average magnetic flux density of the GMM surface is explored. The thickness of the upper conducting magnetic block is 2~9 mm, the thickness of the lower conducting magnetic block is 2~9 mm and the step size is 1 mm. The thickness of the upper conducting magnetic block and the thickness of the lower conducting magnetic sheet are combined and processed in Origin 2024b, and the following figure can be obtained.

**Figure 15:**
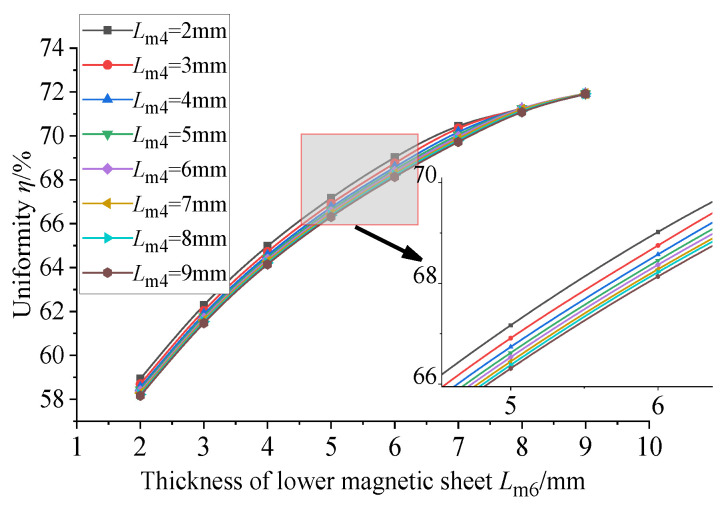
Uniformity under the influence of different thicknesses of upper and lower magnetic blocks.

**Figure 16:**
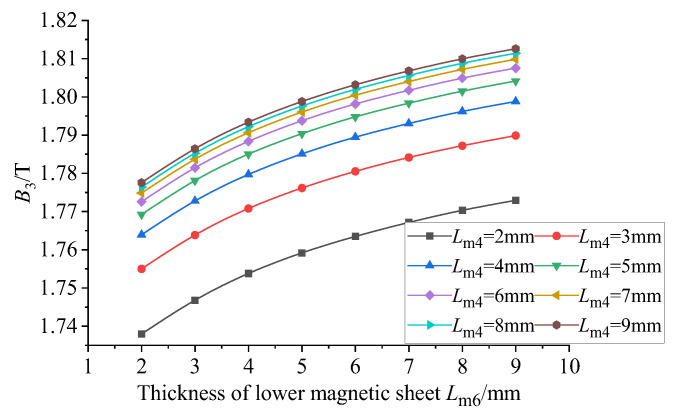
Magnetic flux density under the influence of different thicknesses of upper and lower magnetic blocks.

It can be seen from Figure 15 that when the thickness of the upper conducting magnetic block is constant, the uniformity of the magnetic flux density of the center line of the GMM rod increases with the increase in the thickness of the lower conducting magnetic block, and the increasing trend gradually slows down. When the thickness of the lower conducting magnetic block is between 2 mm and 8 mm, the uniformity decreases with the increase in the thickness of the current magnetic block. When the thickness of the current conducting magnetic block is between 8 mm and 9 mm, although the thickness of the upper conducting magnetic block is different, the uniformity tends to be the same. It can be seen from Figure 16 that when the thickness of the current conducting magnetic block is constant, the average magnetic flux density on the surface of the GMM rod increases with the increase in the thickness of the upper conducting magnetic block, and the increasing trend gradually slows down. When the thickness of the upper conducting magnetic block is 8 mm and 9 mm, the average magnetic flux density is basically the same. When the thickness of the upper conducting magnetic block is constant, the average magnetic flux density on the surface of the GMM rod increases with the increase in the thickness of the lower conducting magnetic block, and the increasing trend gradually slows down. Therefore, when the thickness of the magnetic material is changed separately, the uniformity is the fundamental basis. The thickness of the upper and lower conducting magnetic blocks is more appropriate within a certain range. In this way, the uniformity can be improved, while the average magnetic flux density on the surface of the GMM rod can be maximized.

**Figure 17:**
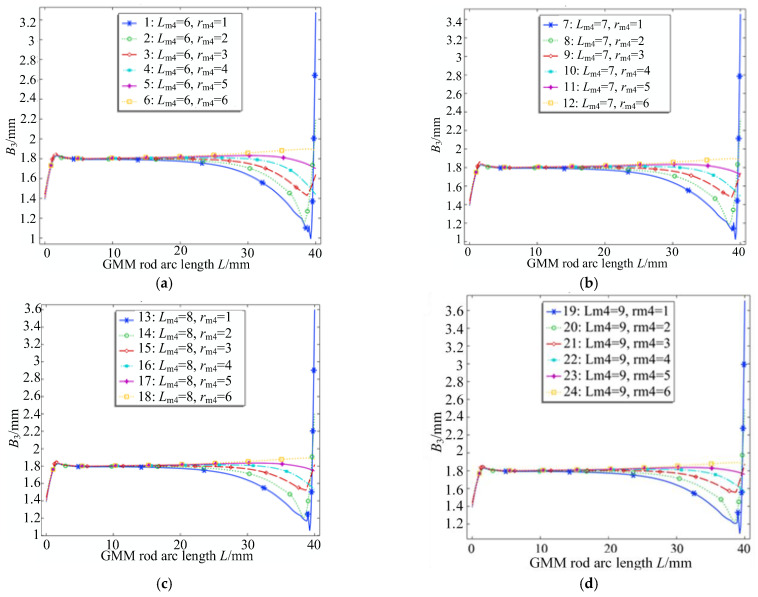
Magnetic flux density of different radii under different thicknesses of upper magnetic block. (**a**) *L*_m4_ = 6 mm; (**b**) *L*_m4_ = 7 mm; (**c**) *L*_m4_ = 8 mm; (**d**) *L*_m4_ = 9 mm.

According to the first equation of the magnetic circuit, the magnetic flux through any closed surface is always equal to the magnetic flux through the closed surface [22]. The thickness of the lower conducting magnetic block is set to 9 mm. Under this condition, the influence of the radius of the upper conducting magnetic block on the flux of the GMM rod is explored. The radius of the upper conducting magnetic block gradually increases, from less than the radius of the GMM rod to greater than the GMM rod. In order to illustrate the general law of the influence of the radius of the upper conducting magnetic block on the magnetic flux of the GMM rod, the upper conducting magnetic blocks with different thicknesses are used. The thickness range of the upper conducting magnetic block is 6~9 mm, the step size is 1 mm, the radius size is 1~6 mm and the step size is 1 mm. The magnetic flux density map under the four thicknesses of (a), (b), (c) and (d) in Figure 17 can be obtained.

**Figure 18:**
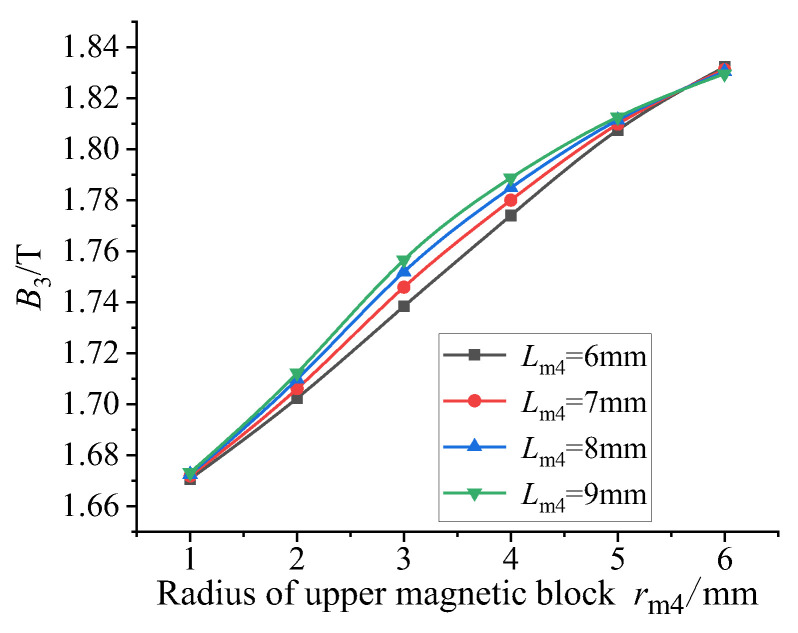
The average magnetic flux density of different radii under different thicknesses of the upper conducting magnetic block.

**Figure 19:**
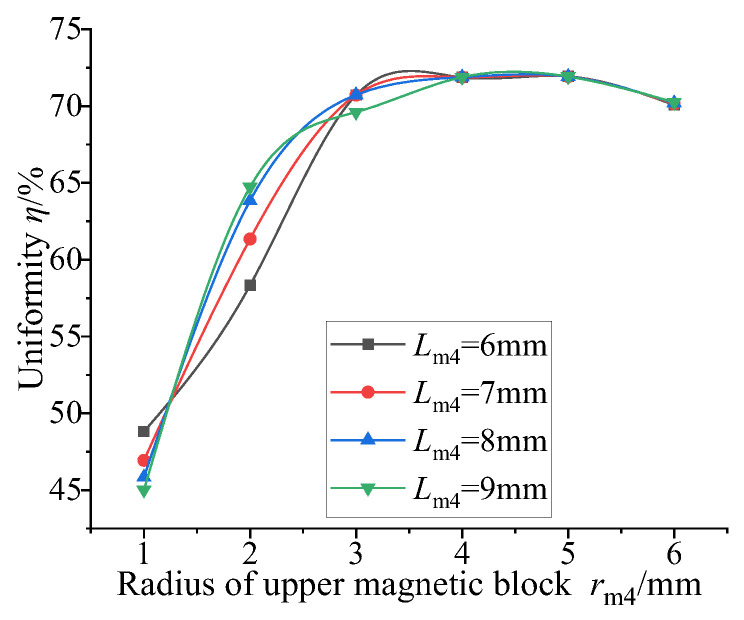
Different radius uniformity under different thicknesses of upper conducting magnetic block.

It can be seen from the four figures shown in Figure 17 that the magnetic flux density at the lower end of the GMM rod is always lower than that at the right end due to the existence of air gap at the lower end of the GMM rod. It can be seen from Figure 18 that when the radius of the upper conducting magnetic block is 1 mm, the average magnetic flux density of the GMM surface is the smallest and the magnetic flux leakage phenomenon is the most serious. As the radius of the upper conducting magnetic block gradually increases, the average magnetic flux density on the surface of the GMM rod also gradually increases, and the magnetic flux leakage phenomenon is gradually improved. It can be seen from Figure 19 that when the radius of the upper conducting magnetic block is 1 mm, the uniformity of the magnetic flux density of the center line of the GMM rod is the smallest; with the increase in the radius of the upper conducting magnetic block, the uniformity of the magnetic flux density increases first and then decreases. When the radius of the upper conducting magnetic block is less than the radius of the GMM rod, the uniformity increases rapidly. The radius of the upper conducting magnetic block is greater than the radius of the GMM rod, and when it is 4~5 mm, the uniformity tends to remain unchanged, and the maximum uniformity appears. The radius of the upper conducting magnetic block continues to increase, and the uniformity shows a trend of slow decline.

Therefore, according to the two factors of uniformity and flux density, the radius of the upper conducting magnetic block should be slightly larger than the radius of the GMM rod when designing the upper conducting magnetic block.

### 4.5. The Influence of Air Gap on the Magnetic Flux of Magnetic Circuit

**Figure 20:**
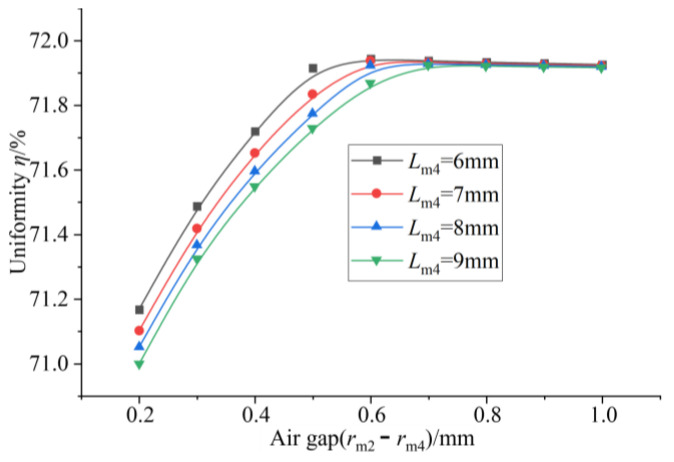
Air gap uniformity under different thicknesses of upper conducting magnetic block.

**Figure 21:**
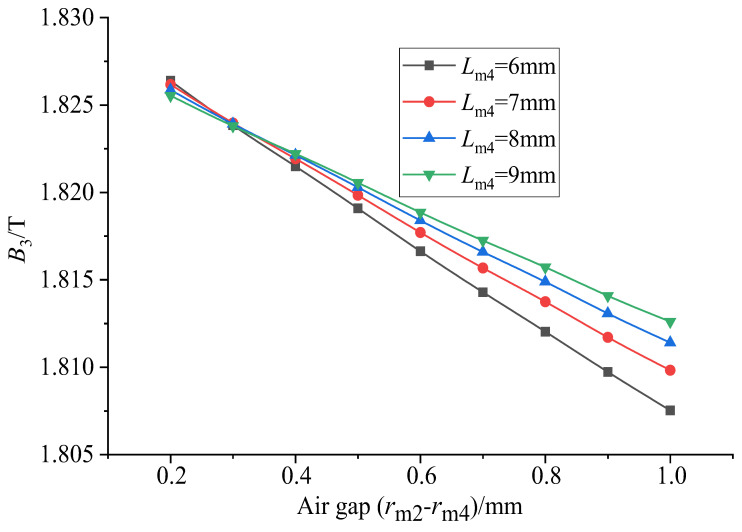
Average value of air gap magnetic flux under different thicknesses of upper conducting magnetic block.

It can be seen from Figure 20 that when the thickness of the upper conducting magnetic block is constant, the uniformity increases first and then tends to remain unchanged with the increase in the air gap. When the air gap is 0.2 mm, the uniformity is the smallest. When the air gap is about 0.7 mm, the uniformity reaches a maximum value. After that, uniformity basically tends to remain unchanged. It can be seen from Figure 21 that the average magnetic flux density on the surface of the GMM rod decreases uniformly with the increase in the air gap, showing a linear change. Therefore, when the air gap is considered separately, the air gap should not be too large, which can not only ensure the uniformity but also improve the magnetic flux density through the GMM rod.

### 4.6. Optimized Combination of Magnetic Circuit Structure

The simulation results show that the above structural parameters have different effects on the internal flux and flux uniformity of the GMM rod. The magnetic flux density uniformity is taken as the result of the orthogonal test, and the optimal combination of the magnetic circuit structure is obtained by the orthogonal experimental factor level analysis method [23,24,25]. In the above simulation, the size of each magnetic circuit structure is used as a separate variable to study its influence on the magnetic flux of the magnetic circuit. In the orthogonal experiment, some of the data are selected as the level of the orthogonal experiment, in which the thickness of the lower conducting magnetic sheet *L*_m9_/mm, the thickness of the upper conducting magnetic block *L*_m4_/mm, the thickness of the lower conducting magnetic block *L*_m6_/mm, the radius of the upper conducting magnetic block *r*_m4_/mm and the air gap (*r*_m4_ − *r*_m2_)/mm are corresponding to factors I, II, III, IV and V. The factor level table is shown in Table 2.

The above factors are independent of each other and are not affected by other variables. The orthogonalization experiment close to the standard orthogonal table L_16_(4^5^) is used for analysis and optimization. The results are shown in Table 3:

Through the orthogonal experiment method, the magnetic circuit structure parameters with the best flux uniformity are *r*_m4_ = 5 mm, *L*_m6_ = 6 mm, *r*_m4_ − *r*_m2_ = 0.8 mm, *L*_m9_ = 1 mm and *L*_m9_ = 4 mm. Considering the cost of production and processing, the lower conducting magnetic sheet is too thin to process. At the same time, through the orthogonal experimental structure, it can be seen that in the N_1_, N_2_, N_3_ and N_4_ corresponding to factor I, N_1_ > N_3_ > N_2_ > N_4_, so the thickness of the lower magnetic sheet is set to 5 mm. At this time, the uniformity of the optimized structure is 67.97%, while the magnetic flux uniformity of the GMM rod in the structure before the combination is 61.19%, which is increased by 6.78%.

### 4.7. Optimization of Giant Magnetostrictive Force Sensor

**Figure 22:**
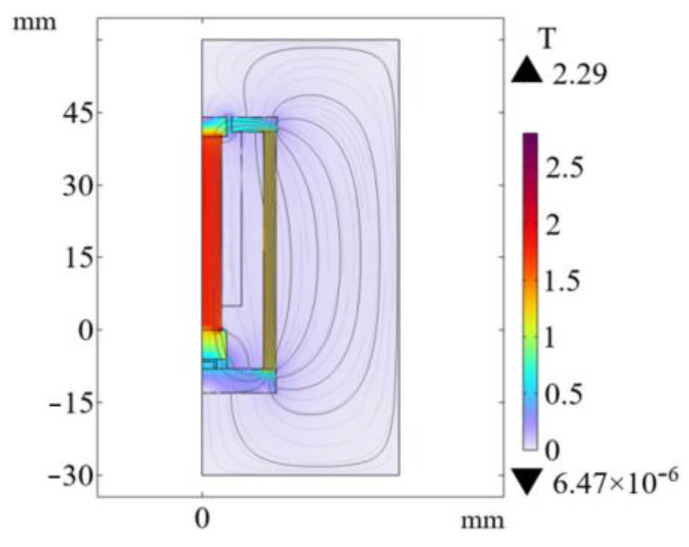
Magnetic circuit diagram of the optimized model.

**Figure 23:**
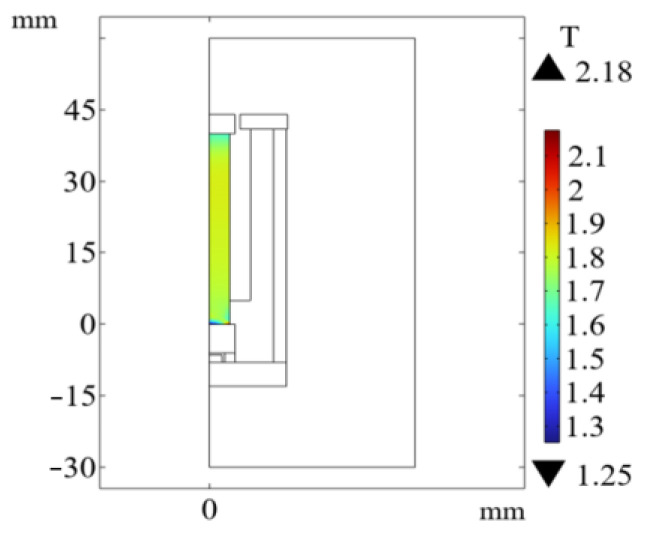
Optimized GMM rod magnetic flux density pattern.

**Figure 24:**
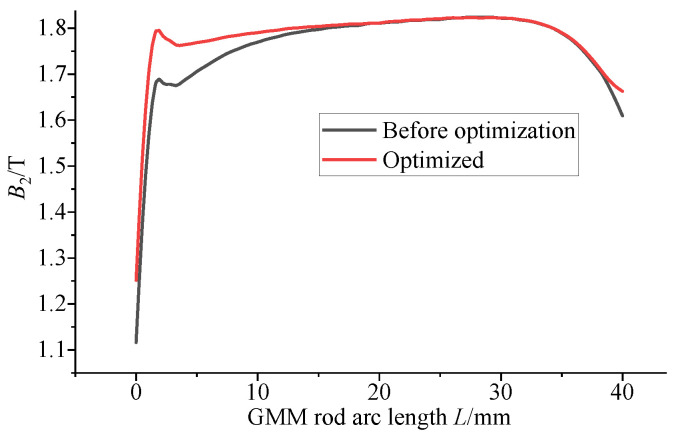
Comparison of magnetic flux density of GMM centerline before and after optimization.

The optimized magnetic circuit adopts the magnetic circuit mode with upper and lower conducting magnetic materials and no magnetic side wall. The radius of the upper conducting magnetic block is 5 mm, the thickness is 4 mm, the thickness of the lower conducting magnetic block is 6 mm, the air gap size is 0.8 mm, the relative permeability of the conducting magnetic material is 4000 and the thickness of the lower conducting magnetic sheet is 5 mm. The magnetic circuit distribution of the optimized model and the magnetic flux density pattern of the GMM rod surface are shown in Figure 22 and Figure 23. The magnetic flux density of the center line of the GMM rod before and after optimization is compared by Origin (2024b), as shown in Figure 24.

It can be seen from Figure 24 that the magnetic induction intensity through the GMM rod has been further improved after optimization, and the magnetic flux distribution is more uniform, and the end effect at both ends of the GMM rod is weakened. Before the structure is not optimized, the uniformity of the magnetic induction intensity of the GMM rod is 61.19%. After the structure is optimized, the uniformity is 68.63%, and the uniformity is increased by 7.44%. Before the optimization, the average surface magnetic induction intensity of the GMM rod is 1.7680 T. After optimization, the average surface magnetic induction intensity of the GMM rod is 1.7879 T, and the magnetic induction is increased by 13.9 mT.

In this study, by optimizing the magnetic field coupling structure, the magnetic flux density acting on the Hall sensing unit is increased from the reference value 1.7680 T to 1.7879 T, and the absolute increment of 19.9 mT is achieved, with a relative increase of 1.125%. According to the Hall effect principle, *V_H_* = *S* · *B*, under the premise that the intrinsic sensitivity *S* of the sensor remains constant, the increase in the magnetic flux density will cause the Hall output voltage *V_H_* to increase linearly in the same proportion.

Its direct effect is to enhance the output signal amplitude, thus effectively improving the signal-to-noise ratio of the system. Theoretical analysis shows that the signal-to-noise ratio is expected to increase by the same proportion under the condition that the power spectral density of the circuit background noise is constant. This optimization reduces the requirements of the amplifier gain and accuracy of the back-end signal conditioning circuit and provides a higher effective resolution for detecting the fine fluctuations of the magnetic field, thus systematically enhancing the overall performance and reliability of the measuring instrument.

## 5. Conclusions

The internal magnetic circuit of the giant magnetostrictive force sensor has a certain complexity. Based on the theory of inverse magnetostrictive effect and the reasonable design of the structure of giant magnetostrictive force sensor, this paper uses COMSOL simulation platform to analyze the magnetic parameters and structure of giant magnetostrictive force sensor by finite element simulation based on the principle of uniformity. The following conclusions can be drawn:

(1) When the permanent magnet is used to provide the bias magnetic field, the uniformity and flux of the GMM rod can be improved by using the non-magnetic side wall. The increase in the relative permeability of the magnetic material can improve the magnetic flux density and magnetic flux uniformity through the GMM rod and reduce the degree of magnetic flux leakage. With the increase in the relative permeability, the magnetic flux density and magnetic flux uniformity through the GMM rod tend to be stable.
(2) The increase in the thickness of the lower conducting magnetic sheet will reduce the magnetic flux density and uniformity through the GMM rod. The increase in the thickness of the upper and lower conducting magnetic block can improve the magnetic flux density and uniformity through the GMM rod. The radius of the upper conducting magnetic block should be slightly larger than the diameter of the GMM rod. The existence of air gap will affect the magnetic flux density intensity and magnetic flux density uniformity through the GMM rod, and the air gap should be set at about 0.8 mm.
(3) The flux density of the optimized giant magnetostrictive force sensor is 13.9 mT higher than that of the GMM rod before optimization, and the uniformity is increased by 7.44%. At this time, the Hall output voltage increases linearly by 1.125%, directly enhancing the output signal amplitude and effectively improving the signal-to-noise ratio of the system.

## Figures and Tables

**Table 1:** Basic parameters of simulation materials.

	GMM	Stainless Steel	Soft Iron	Air	Permanent Magnet
Young’s modulus (Pa)	6 × 10^10^				
Poisson ratio (1)	0.45				
Density (kg/m^3^)	9250		7900		
Electric conductivity (S/m)	5.96 × 10^6^	1.73913 × 10^6^	0	0	
Relative dielectric constant (1)	1	1	1	1	1
Saturation magnetization (A/m)	1.5 × 10^6^				
Saturation magnetostriction (ppm)	1500				
Relative permeability (1)	B–H curve	1	B–H curve	1	
Recoverable permeability (1)					1.05
Remanence (T)					1.2
Relative permeability (1)					1.05

**Table 2:** Factor level table.

Level	Factor				
	Ⅰ	Ⅱ	Ⅲ	Ⅳ	Ⅴ
1	1	2	2	1	0.2
2	3	4	4	3	0.4
3	5	6	6	5	0.6
4	9	9	9	6	0.8

**Table 3:** Orthogonal experiments and results.

Numbering	Ⅰ	Ⅱ	Ⅲ	Ⅳ	Ⅴ	Result
1	1(1)	1(2)	1(2)	1(1)	1(0.2)	42.42%
2	1(1)	2(4)	2(4)	2(3)	2(0.4)	64.97%
3	1(1)	3(6)	3(6)	3(5)	3(0.6)	68.81%
4	1(1)	4(9)	4(9)	4(6)	4(0.8)	70.12%
5	2(3)	1(2)	2(4)	3(5)	4(0.8)	64.85%
6	2(3)	2(4)	1(2)	4(6)	3(0.6)	55.47%
7	2(3)	3(6)	4(9)	1(1)	2(0.4)	50.01%
8	2(3)	4(9)	3(6)	2(3)	1(0.2)	63.82%
9	3(5)	1(2)	3(6)	4(6)	2(0.4)	66.97%
10	3(5)	2(4)	4(9)	3(5)	1(0.2)	70.73%
11	3(5)	3(6)	1(2)	2(3)	4(0.8)	58.25%
12	3(5)	4(9)	2(4)	1(1)	3(0.6)	43.70%
13	4(8)	1(2)	4(9)	2(3)	3(0.6)	54.30%
14	4(8)	2(4)	3(6)	1(1)	4(0.8)	54.28%
15	4(8)	3(6)	2(4)	4(6)	1(0.2)	60.77%
16	4(8)	4(9)	1(2)	3(5)	2(0.4)	56.20%
K_1_	246.32%	228.55%	212.34%	189.75%	237.74%	
K_2_	234.15%	244.78%	234.29%	241.34%	238.15%	
K_3_	239.66%	237.85%	253.22%	260.58%	222.29%	
K_4_	224.88%	233.83%	245.16%	253.33%	246.83%	
N_1_	61.58%	57.14%	53.09%	47.44%	59.44%	
N_2_	58.54%	61.20%	58.57%	60.34%	59.54%	
N_3_	59.91%	59.46%	63.30%	65.15%	55.57%	
N_4_	56.22%	58.46%	61.29%	63.33%	61.71%	
R	5.36%	4.06%	10.22%	17.71%	6.14%	
Secondary factors	Ⅳ, Ⅲ, Ⅴ, Ⅰ, Ⅱ					
Superior level	Ⅳ_3_, Ⅲ_3_, Ⅴ_4_, Ⅰ_1_, Ⅱ_2_					

The meaning of K_1_, K_2_, K_3_ and K_4_ in the orthogonal test table is as follows: K_1_ is the sum of *η* corresponding to the level number l of the corresponding position in I, III, IV and V, and K_2_, K_3_ and K_4_ are analogized in turn. The meanings of N_1_, N_2_, N_3_ and N_4_ in the orthogonal test table are the arithmetic mean values of the occurrence times of the horizontal numbers l, 2, 3 and 4, corresponding to the positions of factors I, II, III, IV and V, where K_l_, K_2_, K_3_ and K_4_ are located. Range R reflects the different degrees of influence of I, II, III, IV and V on the experimental results. The greater the result, the greater the influence of the corresponding factors. The value is the difference between the maximum and minimum values of N_1_, N_2_, N_3_ and N_4_ in each column [26]. Therefore, it can be judged that the radius of the upper conducting magnetic block has the greatest influence on the magnetic flux uniformity of the GMM rod, followed by the thickness of the upper conducting magnetic block, the air gap, the thickness of the lower conducting magnetic sheet and the thickness of the upper conducting magnetic block.

## Data Availability

The original contributions presented in this study are included in the article. Further inquiries can be directed to the corresponding author.
